# Pipeline for transferring annotations between proteins beyond globular domains

**DOI:** 10.1002/pro.4655

**Published:** 2023-07-01

**Authors:** Elizabeth Martínez‐Pérez, Mátyás Pajkos, Silvio C. E. Tosatto, Toby J. Gibson, Zsuzsanna Dosztanyi, Cristina Marino‐Buslje

**Affiliations:** ^1^ Bioinformatics Unit Fundación Instituto Leloir/IIBBA Buenos Aires Argentina; ^2^ Structural and Computational Biology Unit European Molecular Biology Laboratory Heidelberg Germany; ^3^ Department of Biochemistry Eötvös Loránd University Budapest Hungary; ^4^ Department of Biomedical Sciences University of Padua Padua Italy

**Keywords:** annotation, DisProt, homology transfer, intrinsically disordered proteins, multiple sequence alignment, ontology terms, orthologous proteins

## Abstract

DisProt is the primary repository of Intrinsically Disordered Proteins (IDPs). This database is manually curated and the annotations there have strong experimental support. Currently, DisProt contains a relatively small number of proteins highlighting the importance of transferring annotations regarding verified disorder state and corresponding functions to homologous proteins in other species. In such a way, providing them with highly valuable information to better understand their biological roles. While the principles and practicalities of homology transfer are well‐established for globular proteins, these are largely lacking for disordered proteins. We used DisProt to evaluate the transferability of the annotation terms to orthologous proteins. For each protein, we looked for their orthologs, with the assumption that they will have a similar function. Then, for each protein and their orthologs, we made multiple sequence alignments (MSAs). Disordered sequences are fast evolving and can be hard to align, therefore, we implemented alignment quality control steps ensuring robust alignments before mapping the annotations. We have designed a pipeline to obtain good‐quality MSAs and to transfer annotations from any protein to their orthologs. Applying the pipeline to DisProt proteins, from the 1731 entries with 5623 annotations, we can reach 97,555 orthologs and transfer a total of 301,190 terms by homology. We also provide a web server for consulting the results of DisProt proteins and execute the pipeline for any other protein. The server Homology Transfer IDP (HoTIDP) is accessible at http://hotidp.leloir.org.ar.

## 1 INTRODUCTION

The structures of around 30% of eukaryotic protein residues have never been determined by experimental techniques (Perdigão et al., 2015), are inaccessible to template‐based modeling and residues cannot be assigned to a Pfam family, a database of families of protein domains grouped by sequence similarity (Mistry et al., 2021). Most of these unmapped proteins or regions are predicted as disordered or compositionally biased according to several methods and databases such as IUPred3 (Erdős et al., 2021), MobiDB (Piovesan et al., 2021), AlphaFold (Jumper et al., 2021), and others.

The primary repository of disorder‐related data of Intrinsically Disordered Proteins (IDPs) is DisProt (Quaglia et al., 2022), a manually curated database, that ensures that each annotation has an experimental support. DisProt is the result of the effort of more than 60 experts. Disordered status is defined at a regional level and the annotations are enriched with functional ontology terms. The Intrinsically Disordered Proteins Ontology (IDPO) collects structural and functional terms specific to IDPs and has been refactored and systematically cross‐referenced with Gene Ontology (GO) (The Gene Ontology Consortium, 2021). Despite the great advantage of being manually curated, DisProt currently contains a relatively small number of proteins. This is because curating annotations in general, and for disordered proteins in particular, is a labor‐intensive and time‐consuming process, and that direct experimental evidence is also available for a limited number of proteins.

This highlights the importance of transferring annotations regarding verified disorder state and corresponding functions to homologous proteins, adding highly valuable information to better understand their biological roles. We focus on orthologous proteins as they are likely to have similar functions while paralogous proteins may or may not have similar functions. Homology transfer annotation is better established for globular proteins. As an example, the transference of domain annotation as defined by PFAM (Mistry et al., 2021), which is a database of domain families, can be transferred straightforwardly. Multidomain proteins have several structure annotations, one for each domain type. The protein function might or might not coincide with the Pfam domains in the protein architecture.

However, the principles for homology transfer for intrinsically disordered regions, which often show larger evolutionary variation, are much less established.

A major problem to be faced when mapping features between homologous protein sequences is the variable quality of the sequences (Meyer et al., 2020). Only a small number of proteins have themselves been directly sequenced. The vast majority of sequenced DNA has been obtained through large‐scale genome sequencing initiatives of variable quality (Scalzitti et al., 2020). Any group of homologous sequences extracted from the protein sequence databases is likely to contain a mix of high‐ and low‐quality entries.

Most multiple sequence alignment (MSA) algorithms carry the assumption that the sequences to be aligned are collinear. When this condition is not met, alignment quality may be impacted (Thompson et al., 2011). If that happens, then misalignment may lead to errors in feature mapping. Therefore, in a sequence comparison pipeline, it makes sense to remove the most obviously problematic sequences early in the procedure. We expect the proteins in DisProt to be high quality since they are actively researched. Therefore they can act as references against which the problematic proteins can be removed from the homology set. Generating high‐quality alignments will then allow annotation transfer with high confidence.

This work has two main goals, on the one hand, to provide the community with a protocol to safely transfer annotations from any protein to their orthologs. On the other hand, to transfer IDPO and GO annotations from DisProt proteins to their orthologs. Finally, we have made a web server to bring the use of this protocol to the general public.

## 2 RESULTS

**FIGURE 1:**
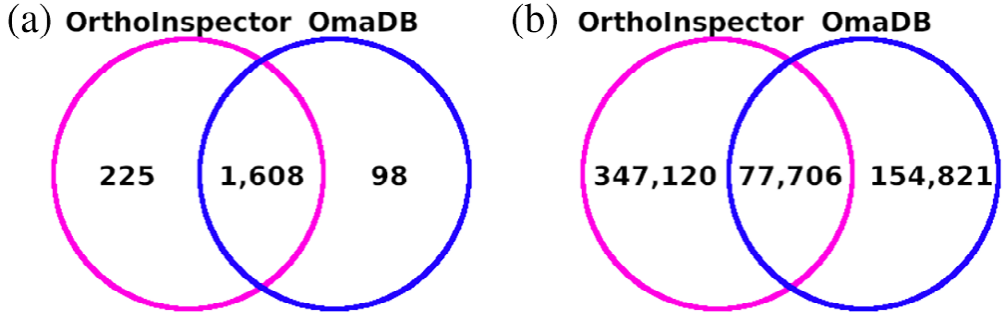
Datasets of orthologous proteins. (a) Venn diagram of DisProt proteins having one‐to‐one orthologs in each database. (b) Venn diagram of one‐to‐one orthologs retrieved from DisProt proteins.

DisProt (Quaglia et al., 2022) is the reference database of IDPs. In the database, disordered regions are enriched with structural and/or functional annotations. In order to test the usability of its information through homology transfer to other proteins, we collected orthologous proteins from two databases, OrthoInspector (Altenhoff et al., 2021) and OmaDB (Nevers et al., 2019). From the 2294 proteins in the DisProt database, only 1931 have available orthologs in these resources (Table S1). A total of 579,647 one‐to‐one orthologous proteins were retrieved from the two databases (Figure 1). As the ortholog databases are different, reference proteins might have orthologs in one database and not in the other. Also, the number of orthologs for a protein in each database can vary. This highlights the importance of using more than one orthology database. Merging them allows us to increase the number of orthologs available to potentially extend the annotations via homology transfer.

However, a key requirement for a reliable homology transfer is a good‐quality MSA. As disorder and corresponding functional annotations are in general assigned to particular segments of the protein, it is also important to examine alignment quality not just at the global level, but also at the level of regions.

### 2.1 Comparison between different parameters

We analyzed different parameters for assigning the suitability of an annotation term to be transferred. The MSAs with each set of parameters and the number of proteins are included in Table 1. The NorMD score was computed for each MSA, a distribution is shown in Figure S1.

**TABLE 1:** Numbers for the 60% and 80% datasets.

Initial DisProt proteins %identity clustering	Alignment method	%identity global MSA	Global MSAs	Number of proteins	%identity region MSA	Region MSAs	Number of proteins
60%	Clustal Omega	> = 60%	1728	128,664	> = 60%	3295	96,571
					> = 80%	3128	70,799
		> = 80%	1621	61,460	> = 60%	3157	57,865
					> = 80%	3064	52,077
	Mafft	> = 60%	1730	134,111	> = 60%	3304	100,753
					> = 80%	3130	72,879
		> = 80%	1628	63,684	> = 60%	3166	60,089
					> = 80%	3070	53,923
**80%**	**Clustal Omega**	**> = 60%**	**1776**	**131,033**	**> = 60%**	**3342**	**99,286**
					> = 80%	3184	72,311
		> = 80%	1674	63,476	> = 60%	3231	59,578
					> = 80%	3135	53,604
	Mafft	> = 60%	1778	137,084	> = 60%	3352	104,250
					> = 80%	3188	74,504
		> = 80%	1682	65,740	> = 60%	3241	61,833
					> = 80%	3143	55,484
Total			13,617	137,528		51,086	104,909

*Note*: Highlighted in bold is the line of work explained in the manuscript for clarity, although all these possibilities were tested, and the results are exposed in the supplementary material.

Statistical differences were found between the NorMD score of the MSAs generated by different sets of parameters (Kruskal–Wallis test, *p* value 1.537349e‐222 < 0.05). To analyze the differences, we made pairwise comparisons between the set of parameters highlighted in Table 1 with the other sets using Dunn's test corrected by False Discovery Rate (see Table S2). As expected, alignments having more than 80% pairwise identity are more accurate based on the NorMD score than alignments at 60% pairwise identity (*p* values <0.05) and there is no dependence either with the initial DisProt percent identity clustering or with the alignment method (*p* values >0.05).

It is worth noting that all the global and region alignments with sequences >60% pairwise identity have a good NorMD score (>0.6).

### 2.2 Suggested annotation transferring for DisProt proteins

If the global alignment has a good score while having a bad score in the annotated region and vice versa, these terms are not suitable to be transferred.

Our initial dataset contained 2294 proteins with 3156 GO terms and 6550 IDPO terms annotated. These proteins resulted in 2151 groups when clustered at 80% identity with CD‐HIT (Fu et al., 2012; Li & Godzik, 2006). Out of these, a total of 1931 sequences had a total of 579,647 one‐to‐one orthologs, ending up with 1849 clusters with more than one entry. For each cluster, we generated MSA with Clustal Omega.

After filtering, a total of 97,555 proteins could be assigned with 301,190 homology transferred terms (84,380 are GO and 220,886 are IDPO terms).

Table 2 shows as an example the regions where GO and IDPO terms could be transferred from the reference protein FUS (UniProt accession P35637) to 53 orthologous proteins in the MSA (alignment NorMD score 1.0). Those orthologs are from 51 organisms: 51 vertebrata (42 mammalia and 17 Primates) and 2 undetermined. Some regions could not be transferred to all the orthologous due to the defined quality threshold (60% identity with FUS).

**TABLE 2:** Example of annotated regions that could be transferred from human FUS protein to the orthologs.

Start	End	NorMD score	Orthologs	ID term	Term name
1	163	1.229	48	GO:0140693	Molecular condensate scaffold activity
				GO:0043232	Intracellular non‐membrane‐bounded organelle
				IDPO:00076	Disorder
1	214	1.000	44	GO:0043232	Intracellular non‐membrane‐bounded organelle
				GO:1990000	Amyloid fibril formation
1	422	1.000	49	GO:0043232	Intracellular non‐membrane‐bounded organelle
1	507	1.000	51	IDPO:00076	Disorder
1	526	1.000	53	GO:0140693	Molecular condensate scaffold activity
				GO:0043232	Intracellular non‐membrane‐bounded organelle
2	36	0.953	48	IDPO:00076	Disorder
37	41	1.000	52	GO:0005515	Protein binding
39	95	1.000	48	GO:1990000	Amyloid fibril formation
98	214	1.000	42	IDPO:00076	Disorder
149	154	0.904	47	GO:0005515	Protein binding
285	370	1.000	52	GO:0003723	RNA binding
				IDPO:00050	Disorder to order
421	455	1.000	52	IDPO:00050	Disorder to order
508	526	1.000	44	GO:0005515	Protein binding

*Note*: FUS is taken as the canonical sequence (UniProt accession P35637).

### 2.3 Evolutionary distance of the transferred regions

In order to examine the evolutionary extent of the annotation transfer from humans, we counted the number of transferred regions at the main eukaryotic evolutionary levels represented by model organisms. Considering the Vertebrate level, the number of transferred annotations was 2045, 1153, and 668 to mouse, chicken, and zebrafish species, respectively. At the Eumetazoa level, represented by Fruitfly and C. elegans, 123 and 18 regions were transferred, respectively. Some of the terms could be transferred to even more distantly related levels. The level of unicellular organisms was represented by yeast with 13 regions, while 14 regions were transferred to the plant‐level model organism, Arabidopsis (see Figure S2). However, this result highly depends on the orthologous source database used for the recruitment. Altogether, the results of the analysis suggest that our method can effectively transfer orthologous regions across a wide range of evolutionary distances, from closely related model organisms to even more distant ones.

### 2.4 Testing homology transfer within DisProt

**FIGURE 2:**
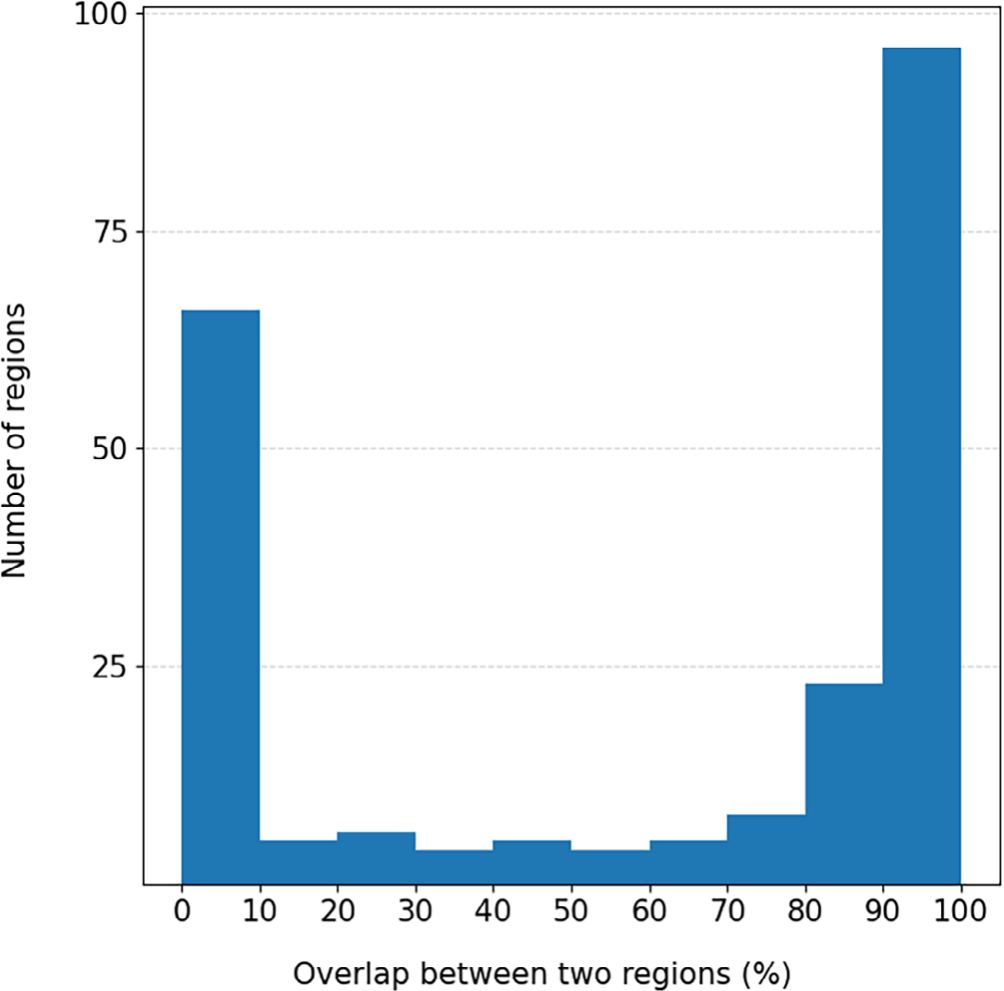
Distribution of overlapped regions between reference and non‐reference proteins.

In total, 222 alignments had more than one protein from DisProt. In 156 cases (70%), the percentage of overlap between annotations was at least 1%. For 61% of the cases (136 cases), the overlap was more than 50% and 54% of the cases (119 cases) the overlap was better than 80%. However, 66 cases (30%) have no overlap between the annotations. The overlap between regions has a double‐peaked distribution (Figure 2).

The absence of overlap, albeit having high sequence identity with the reference protein in the region, is expected because the annotations do not ensure that the authors of the respective original research articles were interested in similar regions/features of the two orthologous proteins.

As a test, for those cases where no annotation was available for the non‐reference DisProt protein, or they do not overlap, we compared the disorder status between the reference and the transferred region, based on AlphaFold (AF) plDDT scores. The analysis was performed on 43 regions of reference proteins (Figure S3). The results show that by applying a 0.7 plDDT score cutoff, the disorder annotation correlates (*p* value <2.2e‐16, rho = 0.98, Spearman rank correlation). However, in more than half of the cases, the experimentally annotated disordered regions were predicted as ordered by AF. This highlights the importance of relying on experimental verification of disorder.

### 2.5 Pipeline to transfer terms by homology

The pipeline for generating high‐quality MSAs and transferring the region annotations is available at https://gitlab.com/idpfun/homology_transfer_disprot. The software requirements are programming languages (Python, R, and Perl), workflow control (snakemake), alignment methods (Clustal Omega and MAFFT), and measuring the MSA quality (norMD score). The pipeline can be downloaded and executed on a personal computer. The default parameters are described in this manuscript and can also be modified in the snake configuration file.

### 2.6 Server HoTIDP homology transfer database

We developed a web server HoTIDP that provides an interface and programmatic access to obtain the transferable terms if a protein has orthologs in DisProt.

The server can be queried with a protein UniProt accession and, if it has an ortholog in DisProt, the annotated regions and transferable terms will be provided. Also, it shows the protein family alignment as well as a pairwise alignment with the reference protein. A table with all the comparison scores (alignment quality of the different regions, and identity % with the reference protein) is also provided. The MSAs can be downloaded and/or visualized.

The default pipeline uses 80% DisProt clustering, Clustal Omega, and global and local alignment at 60% of identity to the reference protein. However, the user can select any of the tested parameters (initial clustering, aligning methods, and the global and local % identity).

It can also suggest transferring a term to any protein if a reference protein and region are provided by the user. The procedure is making an MSA with good quality global and local alignment scores with the orthologous proteins. The server is hosted at http://hotidp.leloir.org.ar.

## 3 DISCUSSION

In this work, we delineated guidelines to transfer annotations to orthologous proteins. In such a way to enrich their functional and structural information, in particular for IDPs. We collected 579,647 one‐to‐one orthologs from 1931 DisProt proteins. We aligned each family of proteins with Clustal Omega and stored the MSAs that surpass a quality threshold. We compared MSAs under different conditions: clustering DisProt proteins by 60% and 80% identity, keeping orthologous sequences by 60% and 80% identity to the reference protein in the global and regional MSAs, and two alignment methods. Our results show that the quality of the alignment does not depend on the alignment method for these similarity cutoffs. As expected, the quality of MSAs constructed gathering sequences more than 80% identical to the reference one, are significantly different (and better) to MSAs made with sequences with more than 60% of identity. However, all MSAs with sequences more than 60% identity also have a good NorMD score.

The MSAs having more than one DisProt protein allowed us to test the method on 222 regions, 136 of which have an overlap of at least 50% of their residues with the reference protein (Figure 2). The overlap between reference and non‐reference protein regions may be underestimated due to differences in the annotation of the two DisProt entries. For instance, the human, mouse, and rat Epsin orthologs are annotated in DisProt; however, the term at human residues 1–18 is not annotated in the closely related orthologs, although these proteins are nearly 100% identical, probably because there is no experimental evidence to support it. This annotation gives important insights into the protein functions, such as that it is disordered, has a transition to an ordered state, and also is a small molecule sequestering region. However, the currently available data limit us to do an exhaustive validation.

Sequences having good NorMD scores in the global alignment (even with high sequence identity) can still have regions with low‐quality scores. If we omit checking the quality of the alignments in the regions, one‐third of the annotations (414,025 terms to 129,257 proteins) would be transferred incorrectly. After filtering sequences in each annotated region of the MSAs, we safely transferred 301,190 terms to 97,555 proteins. This highlights the importance of having this protocol instead of transferring, like other methods do, just considering the identity of the global alignment (Piovesan et al., 2021).

This protocol increases by ~40 times the amount of disordered proteins with functional annotations, and ~25 times the annotated regions with GO terms and ~ 32 times with IDPO terms.

A limitation of our pipeline is to transfer terms that depend on a specific amino acid composition, such as the phosphorylation site that, though having a high identity % in the region, if there is not a serine, threonine, or tyrosine, the phosphorylation will not occur. We identified a total of 11 terms from the DisProt ontology (IDPO) as “tricky cases,” for that homology transferring terms should be taken with care and turned to expert knowledge. These terms, mostly post‐translational modifications, range from IDPO:00024 to IDPO:00034: molecular recognition, phosphorylation, acetylation, methylation, glycosylation, ubiquitination, fatty acylation, myristoylation, palmitoylation, limited proteolysis, and ADP‐ribosylation display sites. These 11 terms, transferred to 6.835 proteins (representing 7% of total orthologs proteins with transferable regions) and 10.476 transferred terms‐regions (representing 3.48% of the total of transferred terms‐regions), should be taken with care.

In summary, we developed guidelines to transfer terms that might give relevant information to homolog proteins.

We also developed a web server HoTIDP that can be used to transfer any term to any protein if the UniProt accession and the regions with the terms are provided.

When we apply the pipeline to the DisProt proteins the GO and IDPO terms annotated can be reliably transferred to more than 97,000 proteins increasing their information.

## 4 MATERIALS AND METHODS

### 4.1 DisProt database

We downloaded version 9.1 (2022–03) of the DisProt (Quaglia et al., 2022) database. It currently collects regions in 2365 proteins with a total of 6763 manually curated IDPO terms and 3251 GO terms. Figure S4 has an example of regions with GO and IDPO terms of protein TP53 potentially suitable to be transferred to its orthologs.

We filtered the dataset considering proteins following the rules: (i) UniProt canonical proteins, (ii) full‐length proteins, (iii) sequences having no undefined amino acids (“X”), and (iv) the protein sequence in DisProt and UniProt are identical. We ended up with 2294 proteins, 3156 GO terms, and 6550 IDPO terms annotated.

### 4.2 Protein dataset

To avoid sequence redundancy, we clustered DisProt proteins with CD‐HIT (Fu et al., 2012; Li & Godzik, 2006), taking the longest one as the reference.

We collected the orthologs for every reference sequence from OmaDB (Altenhoff et al., 2021) and OrthoInspector (Nevers et al., 2019). We then added one‐to‐one orthologous proteins (the relationship between the pair of orthologs) for each reference sequence. Choosing one‐to‐one orthologs decreases the possibility of adding paralogs to the alignments. The sets of proteins obtained from the two databases were merged. Sequences having less than 30% coverage or more than 30% of the length of the reference one, were discarded. Also, all the clusters with only one sequence were removed.

### 4.3 Multiple sequence alignments

**FIGURE 3:**
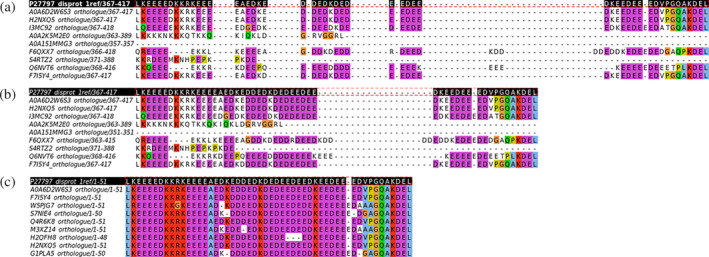
Example of the MSA of CALR, UniProt accession P27797, and DisProt ID DP00333. The region containing residues 367‐417 is annotated in DisProt with the term “disorder” (IDPO:00076) (black row at top of the MSA). Only 10 sequences of the full alignment are shown so gap‐only columns are present in this figure (a) Global MSA with 254 sequences has a bad NorMD score (0.496). (b) Global MSA after eliminating sequences less than 60% identity to the reference one (170 sequences), has a good global NorMD score (0.848) but this particular region still has a bad NorMD score (0.251). (c) Region (DisProt ID DP00333r007) MSA after eliminating sequences less than 60% identity in this region (50 sequences) now has a good NorMD score (1.000). MSA images were processed with Jalview (Waterhouse et al., 2009).

Each cluster with their orthologs was aligned with Clustal Omega (Sievers & Higgins, 2018) and MAFFT (Nakamura et al., 2018). We also tested two sequence alignment conditions: sequences with less than 60% and 80% identity to the reference one (respectively) were removed from the MSAs. Figure 3 shows an example MSA of Calreticulin (CALR) and its orthologs where removing sequences with less than 60% identity to the reference, changes a bad (Figure 3a) into a good MSA (Figure 3b) as measured with NorMD.

Then, we chopped the global alignment and aligned each region containing a term separately. Here, also, we tested removing sequences with less than 60% and 80% identity to the reference one in the aligned region. Figure 3c shows the annotated region (IDPO:00076) aligned after removing sequences with less than 60% identity in the region.

We evaluated the quality of each MSA with the NorMD (Thompson et al., 2001) score. The advantage of the NorMD score is the combination of the column scoring and the residue similarity scores. Additionally, NorMD includes ab initio sequence information, like the amount, length, and similarity of the sequences to be aligned. So, the NorMD score gives information about the general quality of the alignment. A NorMD score >0.6 is considered to indicate a reliable MSA (Muller et al., 2010).

Note that proteins in DisProt can have overlapping annotations which might differ partially because region boundaries sometimes are defined by the construct used in the corresponding experiment. Therefore, the alignment corresponding to each annotation is evaluated separately. The exact boundary of the regions can influence which sequences are removed from the alignment of a particular region and influence which terms are transferred to other sequences.

### 4.4 Testing homology transfer within DisProt

We took advantage of the fact that some alignments have more than one DisProt protein (the reference and non‐reference ones) with annotated GO and IDPO region terms. Using these other members, we tested whether the annotation that we transferred to the non‐reference sequence overlaps with the actual one annotated in the non‐reference proteins. The overlap of a given region was calculated as the percentage of aligned amino acids annotated with a GO or IDPO term between the reference and the non‐reference protein region. In the cases where the aligned region of a given non‐reference protein is not annotated in DisProt, the overlap is zero, even if the identity between the regions is very high (these cases fall into the 0–10 bin). The same applies at different % overlap for regions that are not annotated in exactly the same positions between the 2 proteins (Figure S5 represents the different situations).

### 4.5 WorkFlow chart

We developed a workflow to test the quality of alignments and consequently, the reliability of annotation transfer (Figure S6) shows the general WorkFlow chart used in this work. Briefly, we took the DisProt entries and clustered them. In parallel, we looked for their orthologous proteins. We then aligned each cluster including their orthologs with Clustal Omega and filtered the sequences based on the sequence identity to the reference one, to avoid redundancy. We considered two quality scores: one corresponding to the global alignment (full protein length) and the other to the annotated regions with GO or IDPO terms such as disorder to order transition, protein binding, etc (an example of terms is in Figure S4).

## AUTHOR CONTRIBUTIONS

**Elizabeth Martínez‐Pérez:** Conceptualization (supporting); data curation (equal); formal analysis (lead); investigation (supporting); methodology (equal); software (lead); supervision (equal); validation (equal); visualization (equal); writing – original draft (equal). **Mátyás Pajkos:** Data curation (equal); formal analysis (equal); methodology (equal); software (equal); validation (equal); visualization (equal). **Silvio C. E. Tosatto:** Conceptualization (equal); funding acquisition (equal); project administration (equal); resources (equal); writing – review and editing (equal). **Toby J. Gibson:** Conceptualization (equal); funding acquisition (equal); investigation (equal); supervision (equal); writing – review and editing (equal). **Zsuzsanna Dosztanyi:** Conceptualization (equal); formal analysis (equal); funding acquisition (equal); supervision (equal); writing – review and editing (equal). **Cristina Marino‐Buslje:** Conceptualization (lead); funding acquisition (lead); investigation (lead); project administration (equal); resources (lead); supervision (lead); writing – original draft (equal); writing – review and editing (lead).

## FUNDING INFORMATION

This work is part of a project that has received funding from the European Union's Horizon 2020 research and innovation program under the “Marie Skłodowska‐Curie grant agreement No. 778247” OR “Supported by the H2020‐MSCA‐RISE project IDPfun‐ GA No. 778247.” Elizabeth Martínez‐Pérez is a Postdoctoral fellow and Cristina Marino‐Buslje is a researcher of the Argentine National Research Council (CONICET).

## Supporting information


**Figure S1.** NorMD scores for MSAs. A: NorMD score in global MSAs. B: NorMD score in region MSAs
**Figure S2.** Distribution of the number of transferred regions from humans in model organisms at the main evolutionary level
**Figure S3.** Correlation between the plDDT score of the reference and transferred annotation in the non‐reference DisProt protein when regions do not overlap
**Figure S4.** Example of regions with GO/IDPO terms for P53
**Figure S5.** Examples of aligned IDR sequence regions
**Figure S6.** Outline of the workflow
**Table S1.** DisProt proteins and their orthologs found in the databases OmaDB and OrthoInspector
**Table S2.** Comparison the NorMD scores between sets of alignments, using the Dunn test and the *p* value are corrected with FDR

## Data Availability

All the data and codes are available at the IDPfun GitLab project https://gitlab.com/idpfun/homology_transfer_disprot. Web Server: Homology Transfer (HoTIDP) is accessible at http://hotidp.leloir.org.ar. The server was implemented with Django, Python, R, Celery, RabbitMQ, Snakemake, Bootstrap 5.0, and MSAViewer (Yachdav et al., 2016) JavaScript.
