# Supplementary material for: Pipeline for transferring annotations between proteins beyond globular domains
Source: Protein Sci. 2023 Jul 1;32(7):e4655. doi: 10.1002/pro.4655 (PMC10278204; doi:10.1002/pro.4655)
Supplement: Supplementary file 1 — Figure S1. NorMD scores for MSAs. A: NorMD score in global MSAs. B: NorMD score in region MSAs Figure S2. Distribution of the number of transferred regions from humans in model organisms at the main evolutionary level Figure S3. Correlation between the plDDT score of the reference and transferred annotation in the non‐reference DisProt protein when regions do not overlap Figure S4. Example of regions with GO/IDPO terms for P53 Figure S5. Examples of aligned IDR sequence regions Figure S6. Outline of the workflow Table S1. DisProt proteins and their orthologs found in the databases OmaDB and OrthoInspector Table S2. Comparison the NorMD scores between sets of alignments, using the Dunn test and the p value are corrected with FDR [file PRO-32-e4655-s001.pdf]

Pipeline for transferring annotations between proteins beyond globular domains

Elizabeth Martínez-Pérez[#,1,2], Mátyás Pajkos[#,3], Silvio C.E. Tosatto[4], Toby J. Gibson[2], Zsuzsanna Dosztanyi[3▲] and Cristina Marino-Buslje[1▲*]

SUPPLEMENTARY MATERIAL

1. Bioinformatics Unit, Fundación Instituto Leloir/IIBBA, Buenos Aires, Argentina.
2. Structural and Computational Biology Unit, European Molecular Biology Laboratory, Heidelberg 69117, Germany
3. Department of Biochemistry, Eötvös Loránd University, Pázmány Péter stny 1/c, Budapest, H-1117, Hungary
4. Department of Biomedical Sciences, University of Padua, Italy.
[#]Contributed equally, both are first authors
[▲] Contributed equally
* Corresponding author cmb@leloir.org.ar

Elizabeth Martínez Pérez (ORCID: 0000-0003-4411-976X) emartinez@leloir.org.ar
Mátyás Pajkos (0000-0001-5791-9825) matyas.pajkos@ttk.elte.hu
Silvio C.E. Tosatto (ORCID: 0000-0003-4525-7793) silvio.tosatto@unipd.it
Toby J. Gibson (ORCID: 0000-0003-0657-5166) toby.gibson@embl.de
Zsuzsanna Dosztányi (ORCID:0000-0002-3624-5937) zsuzsanna.dosztanyi@ttk.elte.hu
Cristina Marino Buslje (ORCID: 0000-0002-6564-1920) cmb@leloir.org.ar

Supplementary Figure 1.

A

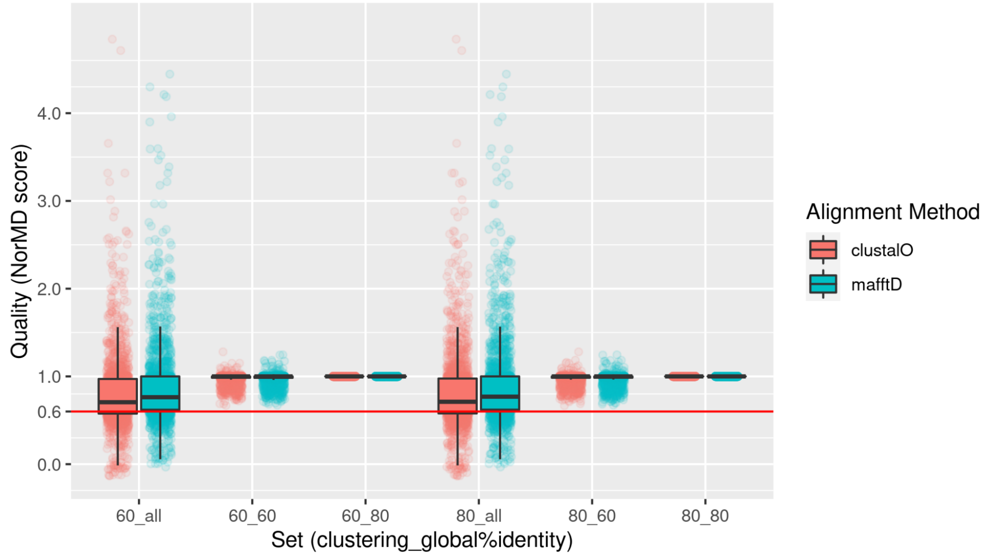

B

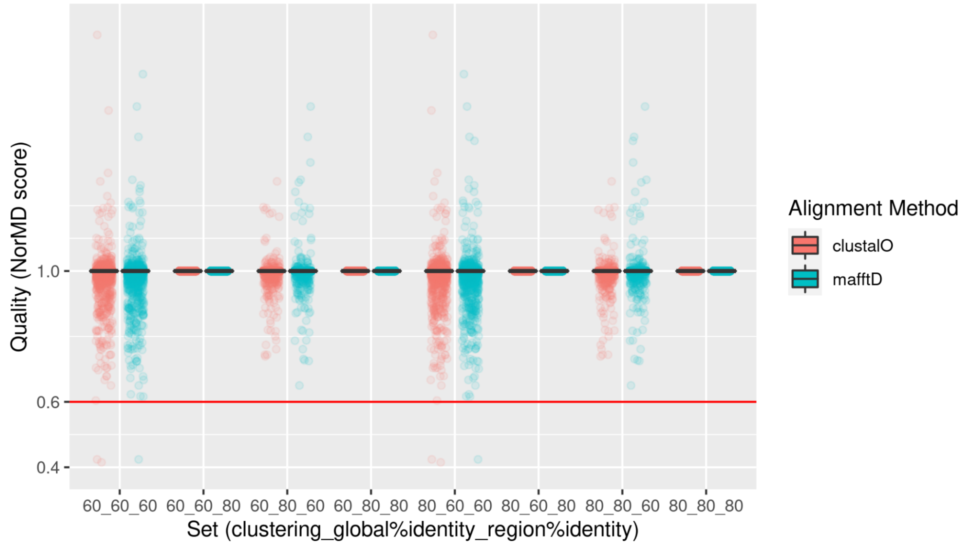

Supplementary Figure 1. NorMD scores for MSAs. A: NorMD score in global MSAs. B: NorMD score in region MSAs. The "y" axis is the NorMD score. The "x" axis are the sets to compare using the nomenclature AA_BB or AA_BB_CC where AA is clustering type, BB the % on the global MSAs and CC is the %identity on the region MSAs. All the %identity mentioned are between each sequence and the reference in the MSA. The BB as "all" are global MSA without filter sequence by %identity.

Supplementary Figure 2.

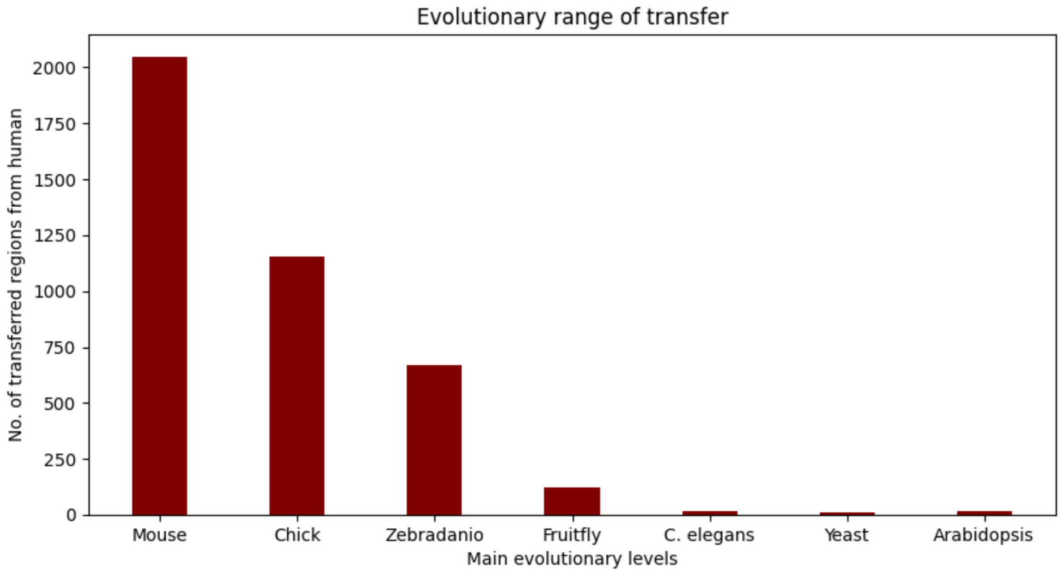

Supplementary Figure 2. Distribution of the number of transferred regions from humans in model organisms at the main evolutionary level.

Supplementary Figure 3.

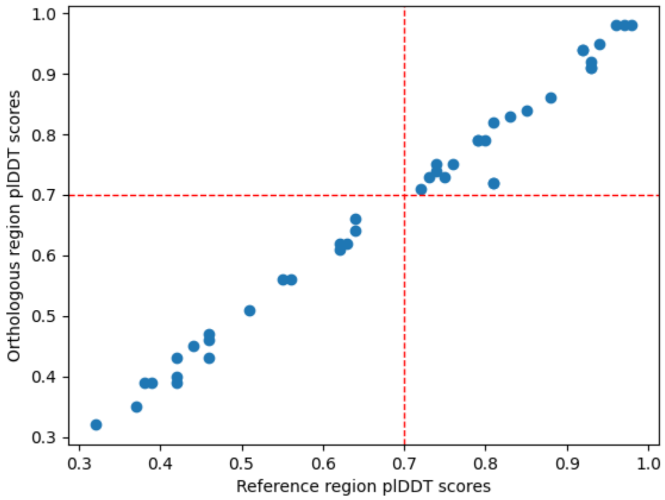

Supplementary Figure 3. Correlation between the plDDT score of the reference and transferred annotation in the non reference DisProt protein when regions do not overlap. The plDDT score is extracted from the PDB files generated by AF. The values were averaged for each region (in the reference and transferred region in the non reference protein of the MSA.

Supplementary Figure 4.

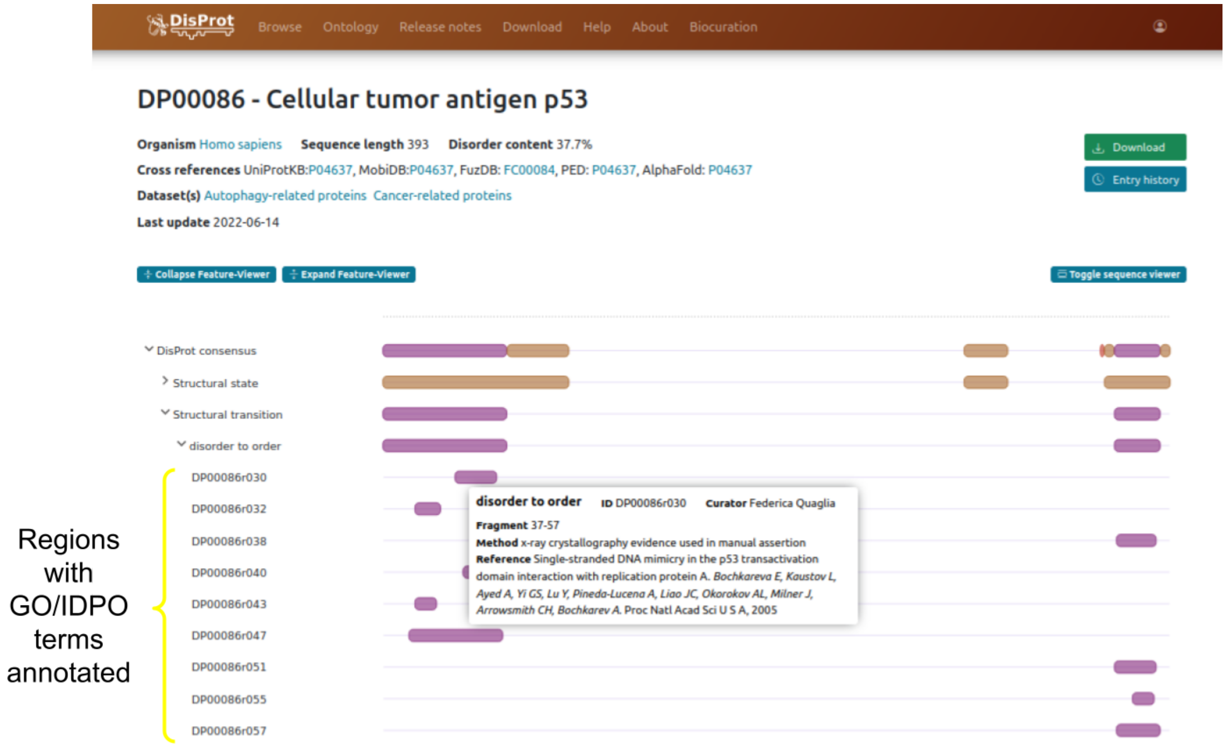

Supplementary Figure 4. Example of regions with GO/IDPO terms for P53. Screenshot showing detail for DisProt accession DP00086. At the top level of the ontology are the Structural state, Structural transition and Disordered function. The other terms are in the second level of the ontology, and the notations like DP00086r030 are regions with the specific IDPO terms.

Supplementary Figure 5.

```
       Reference: MASNDYTQQATQSYGAYPTQPGQGYSQQSSQPYGQQSYSGYSQSTDTSGYGQSSYSSYGQ0
A 100% overlap: MASNDYTQQATQSYGAYPTQPGQGYSQQSSQPYGQQSYSGYSQSTDTSGYGQSSYSSYGQ0
B 100% overlap: MASNDYTQQATQSYGAYPTQPGQGYSQQSSQPYGQQSYSGYSQSTDTSGYGQSSYSSYGQ0
C  50% overlap: MASNDYTQQATQSYGAYPTQPGQGYSQQSSQPYGQQSYSGYSQSTDTSGYGQSSYSSYGQ0
D  50% overlap: M-----------------TQPGQGYSQQSSQPYGQQSYSGYSQSTDTSGYGQSSYSSYGQ0
E   0% overlap: MASNDYTQQATQSYGAYPTQPGQGYSQQSSQPYGQQSYSGYSQSTDTSGYGQSSYSSYGQ0
F   0% overlap: M-----------------------------------SYSGYSQSTDTSGYGQSSYSSYGQ0
```

Supplementary Figure 5. Examples of aligned IDR sequence regions. The Reference sequence is FUS (UniProt Accession P35637) amino acids 1-60. The orange box shows the range of the annotated "disorder" (term IDPO:00076) for amino acids 2 to 36. The sequences A-F and their annotations are artificial examples. 0% overlap means no annotation of this region in the other DisProt protein (sequence E) or a region not present in the orthologue (Sequence F).

Supplementary Figure 6.

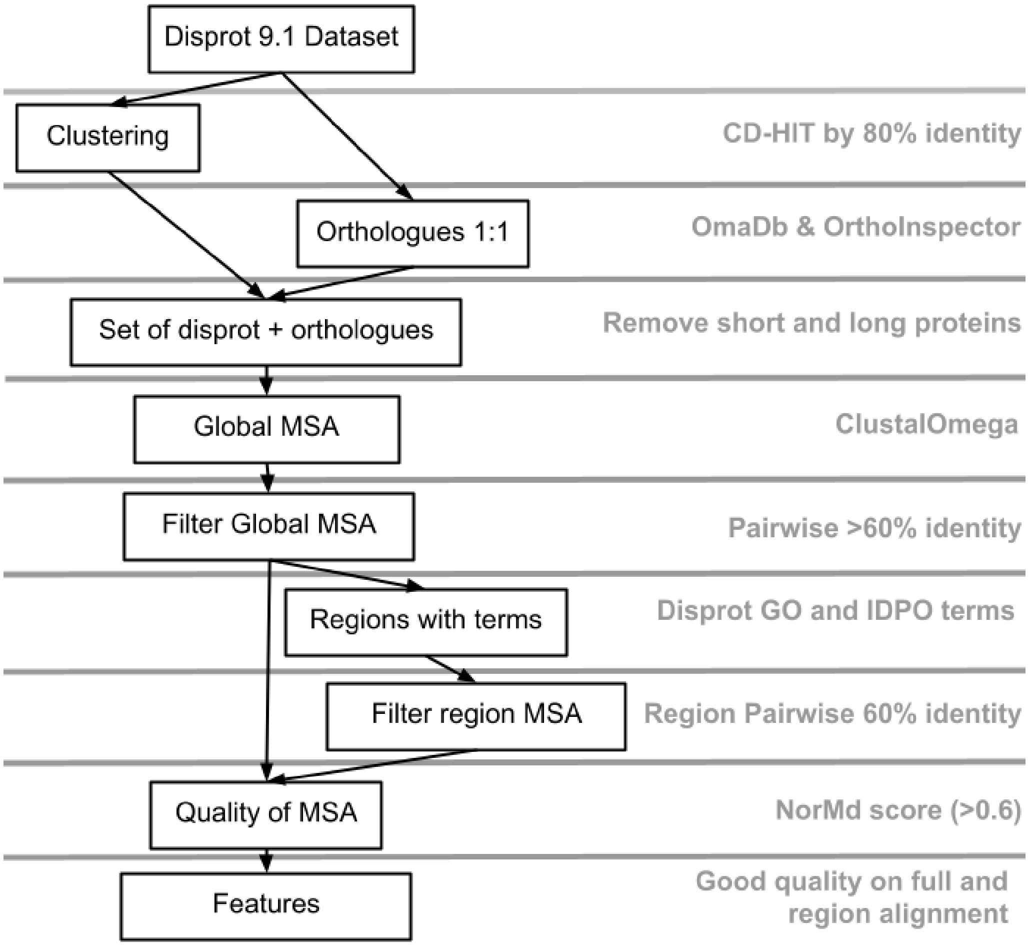

Supplementary Figure 6. Outline of the workflow. Reference DisProt sequences are used to collect orthologs and produce MSAs. After quality evaluation, annotated features are transferred.

Supplementary Table 1. DisProt proteins and their orthologs found in the databases OmaDB and OrthoInspector.

	OmaDB	OrthoInspector	Total
DisProt proteins not found	509	429	
DisProt proteins found with valid one-to-one orthologs	1,697	1,827	1,924
Valid orthologs found	230,997	424,030	577,653

We consider valid orthologues when the protein is: not obsolete in UniProt, has a Complete sequence (no fragments), and has no "X" in the primary sequence.

Supplementary Table 2. Comparison of the NorMD scores between sets of alignments, using the Dunn test and the p-value are corrected with FDR.

Disprot clustering	Alignment method	Global %identity +	Region %identity +	P-value comparing with Default parameters*
60	Clustal Omega	60	60	**8.39E-01**
60	Clustal Omega	60	80	3.41E-43
80	Clustal Omega	60	80	3.41E-43
60	Clustal Omega	80	60	1.91E-15
60	Clustal Omega	80	80	7.16E-43
80	Clustal Omega	80	60	1.89E-15
80	Clustal Omega	80	80	3.41E-43
60	MAFFT	60	60	**4.50E-01**
60	MAFFT	60	80	3.41E-43
80	MAFFT	60	60	**4.50E-01**
80	MAFFT	60	80	3.41E-43
60	MAFFT	80	60	2.28E-18
60	MAFFT	80	80	7.16E-43
80	MAFFT	80	60	7.85E-19
80	MAFFT	80	80	3.41E-43

+ %identity is the pairwise value between each sequence and the reference sequence

*** Default parameter** is DisProt clustering 80% identity, Clustal Omega as alignment method, 60% identity in global and region Pairwise alignment with the reference protein.
